# A Comparison of ChatGPT and Fine-Tuned Open Pre-Trained Transformers (OPT) Against Widely Used Sentiment Analysis Tools: Sentiment Analysis of COVID-19 Survey Data

**DOI:** 10.2196/50150

**Published:** 2024-01-25

**Authors:** Juan Antonio Lossio-Ventura, Rachel Weger, Angela Y Lee, Emily P Guinee, Joyce Chung, Lauren Atlas, Eleni Linos, Francisco Pereira

**Affiliations:** 1 National Institute of Mental Health National Institutes of Health Bethesda, MD United States; 2 School of Medicine University of Pittsburgh Pittsburgh, PA United States; 3 Department of Communication Stanford University Stanford, CA United States; 4 National Center For Complementary and Alternative Medicine National Institutes of Health Bethesda, MD United States; 5 School of Medicine Stanford University Stanford, CA United States

**Keywords:** sentiment analysis, COVID-19 survey, large language model, few-shot learning, zero-shot learning, ChatGPT, COVID-19

## Abstract

**Background:**

Health care providers and health-related researchers face significant challenges when applying sentiment analysis tools to health-related free-text survey data. Most state-of-the-art applications were developed in domains such as social media, and their performance in the health care context remains relatively unknown. Moreover, existing studies indicate that these tools often lack accuracy and produce inconsistent results.

**Objective:**

This study aims to address the lack of comparative analysis on sentiment analysis tools applied to health-related free-text survey data in the context of COVID-19. The objective was to automatically predict sentence sentiment for 2 independent COVID-19 survey data sets from the National Institutes of Health and Stanford University.

**Methods:**

Gold standard labels were created for a subset of each data set using a panel of human raters. We compared 8 state-of-the-art sentiment analysis tools on both data sets to evaluate variability and disagreement across tools. In addition, few-shot learning was explored by fine-tuning Open Pre-Trained Transformers (OPT; a large language model [LLM] with publicly available weights) using a small annotated subset and zero-shot learning using ChatGPT (an LLM without available weights).

**Results:**

The comparison of sentiment analysis tools revealed high variability and disagreement across the evaluated tools when applied to health-related survey data. OPT and ChatGPT demonstrated superior performance, outperforming all other sentiment analysis tools. Moreover, ChatGPT outperformed OPT, exhibited higher accuracy by 6% and higher *F*-measure by 4% to 7%.

**Conclusions:**

This study demonstrates the effectiveness of LLMs, particularly the few-shot learning and zero-shot learning approaches, in the sentiment analysis of health-related survey data. These results have implications for saving human labor and improving efficiency in sentiment analysis tasks, contributing to advancements in the field of automated sentiment analysis.

## Introduction

### Background

Sentiment analysis is a field within natural language processing (NLP) that aims to extract sentiments and opinions from text related to specific entities and topics [1], such as people, organizations, events, and places [2]. Specifically, we consider the task of classifying texts as positive, neutral, or negative. Research in this area can occur at different levels of granularity, ranging from a single sentiment for an entire document or for each sentence within it to exploring various aspects associated with each entity, which can be associated with different sentiments [1,3].

Recently, we have witnessed an increase in the use of sentiment analysis to computationally evaluate the attitudes, perceptions, and emotions of social media users regarding the COVID-19 pandemic [4,5]. Most of these works study content from social media platforms such as Twitter, Reddit, and Facebook [6], as social media has been a main platform to express opinions related to COVID-19 in a public manner. Simultaneously, surveys, which refer to data collected from a group of people regarding their opinions, behavior, or knowledge through specifically designed questions, have also been used to investigate the impact of the COVID-19 pandemic. In particular, surveys conducted during the lockdown period in 2020 examined the effects on people’s lives, behaviors, and mental health, among other topics [7-9]. Web-based surveys are often semistructured, that is, composed of closed-answer components (eg, different clinical questionnaires) and open-ended questions that allow a free-text answer. Sentiment analysis tools have been applied to the latter to help monitor the attitudes, sentiments, and perceptions of the participants during the pandemic to assist health decision-making [10].

The application of sentiment analysis tools on free-text data obtained from surveys poses challenges for health care providers and researchers in the health domain. This is partly attributed to the fact that most state-of-the-art applications are designed for different domains, such as social media, and there is limited knowledge regarding their performance in survey data. In addition, recent studies have applied the most well-known sentiment analysis tools, including TextBlob [11], VADER (Valence Aware Dictionary and Sentiment Reasoner) [12], and Stanza [13], to analyze health-related content on social media platforms [14-16] and, more recently, in the context of COVID-19 [6,17]. These studies highlighted the need for a more comprehensive evaluation of sentiment analysis tools, as the initial results exhibited a lack of accuracy and yielded inconsistent outcomes [15,16]. The main reason for this discrepancy was the disparity in data sets and the potential sensitivity of the tools to the composition of the data set [16]. Consequently, researchers trained new algorithms tailored to their specific data set.

Two COVID-19 survey data sets were used in this study, both collected by teams from the National Institutes of Health (NIH) and Stanford University. The collected data were used to assess the general topics experienced by the participants during the pandemic lockdown.

Researchers from both institutions aimed to comprehend the general sentiment patterns over time and identify an overall sentiment for events during that period, such as vaccines and the 2020 presidential elections. In both data sets, it was often the case that a complete response contained multiple topics, with many sentences referring to distinct subjects. Thus, this study is focused on the analysis of sentiment at the sentence level. By assessing each sentence independently, subtle shifts in sentiment could be captured, which could potentially be neglected at the document level. Moreover, we thought that an analysis based on sentence level, rather than aspect-based level, was more appropriate, given that our focus was not on the granularity of the various aspects of an entity. For instance, when evaluating different features of an intensive care unit, aspects might encompass ventilators, rooms, staff, nurses, and others. Therefore, the decision to focus on sentence-level sentiment analysis is influenced by practical considerations, our research objectives, and the nature of the survey responses.

In this study, as the first contribution, we analyzed 2 independent survey data sets containing free-text data collected during the lockdown period of the COVID-19 pandemic, with accompanying ground-truth sentiment labels generated by human raters for hundreds of responses. The second contribution involves a comparison of 8 widely used state-of-the-art sentiment analysis tools, which have been frequently and recently used in the health domain [16], on COVID-19 surveys at the sentence level. We demonstrate that performance across tools varies and that there is a complex correlation structure between their predicted polarity scores. The third contribution of this paper is to investigate whether the polarity prediction performance can be improved through few-shot learning on a small labeled data set or zero-shot learning with ChatGPT [18].

### Related Work

There are 2 main approaches to performing sentiment analysis: lexicon based and machine learning based. Initial lexicon methods are the simplest rule-based methods and seek to classify the sentiment of a sentence as a score function of the word polarities existing in a dictionary [19-23]. Lexicon-based techniques use mostly adjectives and adverbs to compute the overall sentiment score of a text, for instance, Linguistic Inquiry and Word Count (LIWC) [24], Affective Norms for English Words [25], and SentiWordNet [26]. Dictionaries of lexicons are created either manually or automatically [27,28]. First, a list is generated from a specific domain. Then synonyms and antonyms are added from other existing dictionaries such as WordNet [29]. More sophisticated lexicon-based methods focus on complex rules, such as regular expressions [30,31], instead of simply computing a sentiment score based on word polarities.

Machine learning–based techniques use statistical methods to compute sentiment polarity. The process involves training a classifier on a labeled data set, such as movie reviews or social media posts, and then using the model to predict the sentiment of new, unlabeled data. Obtaining labeled data to train the classifiers is a time-consuming task. Machine learning–based methods often face challenges when processing negative and intensifying statements and can have low performance when applied to different domains, as they rely mainly on the data set size. The rules proposed in the lexicon-based approaches have also been used to extract relevant features and used as input to machine learning algorithms (eg, naive Bayes, *k*-nearest neighbors, decision tree, and logistic regression) to predict the sentiment [32-37]. Other machine learning methods are based on deep neural networks (DNNs). DNNs have been successfully used for sentiment analysis, as described in detail by Birjali et al [3], Zhang et al [38], and Yadav and Vishwakarma [39], having achieved state-of-the-art performance on several benchmarks. DNN architectures used include recurrent neural networks [40,41], long short-term memory networks [42,43], and convolutional neural networks [44-46].

More recently, transformers [47] (deep learning architectures) and large language models (LLMs) have gained popularity due to their ability to perform NLP tasks, including sentiment analysis, with remarkable performance. These LLMs have been pretrained on large text corpora using transformers, such as Bidirectional Encoder Representations from Transformers (BERT) [48], Robustly optimized BERT approach (RoBERTa) [49], Embeddings from Language Models (ELMo) [50], Generative Pre-trained Transformers (GPT) [51], and Pathways Language Model (PaLM) [52]. LLMs in sentiment analysis can handle several data types and domains as well as identify patterns and relationships between the semantics of words and phrases that are indicative of sentiment. LLMs for sentiment analysis can also be fine-tuned to specific domains and applications, which usually lead to better results, as shown in previous studies [53-59]. Finally, ChatGPT (OpenAI) [18] has suddenly emerged to produce human-like responses to user inputs. The notable performance of LLMs has led to increased interest in few-shot and zero-shot learning methods using them. Few-shot learning algorithms enable a model to learn from only a few examples, whereas zero-shot learning algorithms can transfer knowledge from one task to another without additional labeled training examples. These approaches have demonstrated comparable or superior performance to prior state-of-the-art fine-tuning methods on various NLP tasks [60-62].

Sentiment analysis has become an increasingly popular technique in the health domain, as noted in the study by Rodríguez-Ibánez et al [63]. A recent study [64] also found that the main data source for studies on health is social media, such as Twitter and Facebook. This is attributed to advancements in mobile technology and their use as a source in health-related topics, such as finding treatments, sharing experiences and opinions, and addressing public health surveillance issues [65-67]. During the pandemic, we witnessed social media becoming the main forum to express opinions related to COVID-19, which helped authorities to understand and monitor sentiments toward topics related to the pandemic [68-73].

Various studies have proposed new sentiment analysis methods and compared existing tools (eg, TextBlob [74], VADER [12], and Stanza [13]) on topics related to COVID-19, mainly extracted from social media [6,16,17,75-78]. However, to the best of our knowledge, there are no studies that have compared several sentiment analysis tools on health-related surveys—a more structured type of text data than social media posts—that collected knowledge, beliefs, and habits during the COVID-19 pandemic [79-84]. The only study we are aware of that evaluates ChatGPT on various sentiment analysis tasks, comparing it with fine-tuned BERT, is the study by Wang at al [85]. The results demonstrated that ChatGPT exhibited promising zero-shot sentiment analysis ability, achieving performance on par with fine-tuned BERT and state-of-the-art models. However, it fell slightly behind domain-specific fully supervised state-of-the-art models.

## Methods

**Figure 1:**
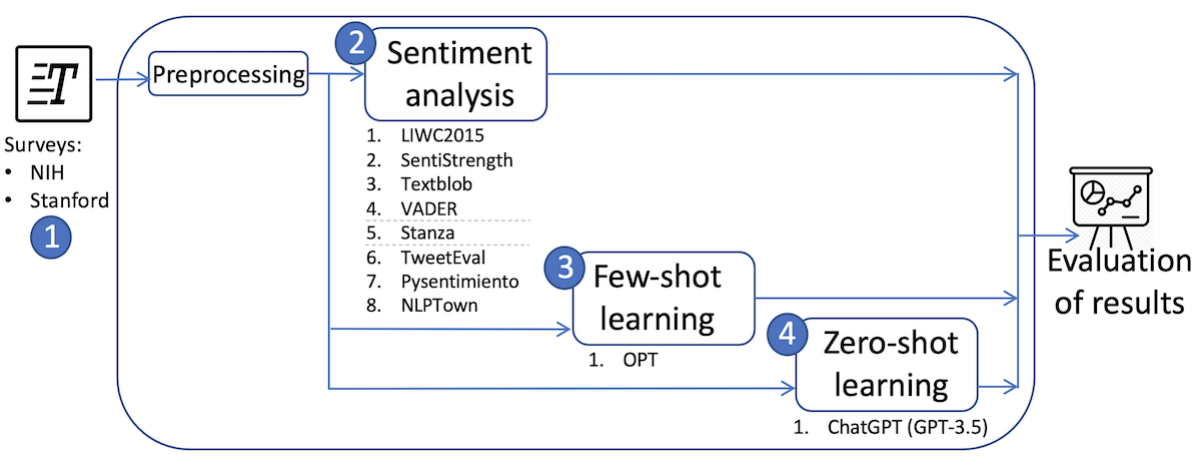
Workflow of our study for evaluating sentence sentiment analysis using state-of-the-art sentiment analysis tools, few-shot learning with a large language model, and zero-shot learning with ChatGPT over health-related surveys. GPT: Generative Pre-trained Transformers; LIWC2015: Linguistic Inquiry and Word Count 2015; NIH: National Institutes of Health; OPT: Open Pre-Trained Transformers; VADER: Valence Aware Dictionary and Sentiment Reasoner.

This section presents the data sets used in this study along with our evaluation of sentence sentiment analysis methods, as illustrated in Figure 1. Specifically, we describe the (1) survey data sets, (2) state-of-the-art sentiment analysis tools, (3) few-shot learning with an LLM, and (4) zero-shot learning with ChatGPT.

### Data

#### NIH Data Set

This data set was collected as part of a web-based survey assessing mental health during the pandemic, which started from April 2020 to May 2021. This was a sample of convenience, as participants were recruited from a pool of previous participants in the National Institute of Mental Health and National Center for Complementary and Alternative Medicine studies by advertising on social media and by flyers within the Washington metropolitan area. Participants who signed up completed various questionnaires at baseline, assessing demographics, clinical history, and psychological state [86]. The participants were then sent emails every 2 weeks for 6 months, inviting them to complete 3 of those questionnaires at that time. This latter survey consisted of 45 questions assessing various attitudes, behaviors, and impacts surrounding the pandemic and a single free-response question (“Is there anything else you would like to tell us that might be important that we did not ask about?”). There was a maximum of 13 potential survey (and free) responses per participant. Of the 3655 participants who enrolled in the study, 2497 (68.31%) responded at least once to the free-response item, yielding a total of 9738 item responses. These were composed of 26,411 sentences, which were the data used in this study. The semantic content of these responses (eg, main topics of concern over time) is available in the study by Weger et al [87].

#### Stanford Data Set

This data set was collected as part of a web-based survey conducted from March to September 2020 by a Stanford University team. The survey was conducted using a sample of convenience recruited through 3 social media platforms: Twitter, Facebook, and Nextdoor. They could participate by clicking on a survey link in the social media post upon seeing the recruitment materials. The survey comprised 21 questions including demographics and the impact of COVID-19 on individuals’ lives [88]. In this study, we focus on the evaluation of 3 free-text responses to the following questions: (1) “Although this is a challenging time, can you tell us about any positive effects or ‘silver linings’ you have experienced during this crisis?” (2) “What are the reasons you are not self-isolating more?” and (3) “Have you experienced any difficulties due to the coronavirus crisis?.” Of the 4582 participants recruited, 3349 (73.09%) responded to at least 1 of the 3 free-text questions, resulting in a total of 7182 item responses. These were composed of approximately 21,266 sentences, which were the data used in this study. The topics and sentiments in these responses are reported in the study by Lossio-Ventura et al [10]. Table 1 presents additional details regarding the NIH and Stanford data sets.

**Table 1:** Details of the National Institutes of Health (NIH) and Stanford data sets.

	NIH	Stanford
Start of the collection period	April 2020	March 2020
End of the collection period	May 2021	September 2020
Responders, n/N (%)	2497/3655 (68.31)	3349/4582 (73.09)
Response items, n	9738	7182
Sentences before processing, n	26,411	21,266
Sentences after processing, n/N (%)	26,188/26,411 (99.16)	21,035/21,266 (98.91)
Tokens after processing, n	462,518	299,735
Tokens per sentence, mean (SD)	17.66 (11.11)	14.25 (9.74)

#### Annotation

We created training and test sets for both the NIH and Stanford data sets. These sets were derived from the surveys after completing the preprocessing steps and were used for training, tuning, and the official evaluation.

##### Training Data Set

We randomly selected 260 sentences, with 130 sentences from each data set. Each subset of 130 sentences was annotated by a different annotator. The annotators were instructed to assign a polarity value of −1 (negative), 0 (neutral), or 1 (positive) to each sentence.

##### Test Data Set

A total of 1000 sentences were randomly chosen, with 500 sentences selected from each data set [89]. Each set was annotated by 3 separate and independent annotators: A.1, A.2, and A.3 for NIH and A.4, A.5, and A.6 for Stanford. The annotators were instructed to assess the polarity of each sentence on a scale of −1 (negative), 0 (neutral), or 1 (positive).

**Figure 2:**
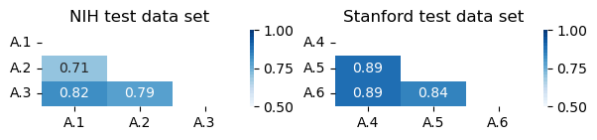
Correlation of annotators on the National Institutes of Health (NIH) and Stanford test data sets. A.1, A.2, A.3 represent the 3 independent NIH annotators, while A.4, A.5, A.6 represent the Stanford annotators.

We used a 3-point scale to annotate the data. We then followed a 3-step procedure to determine the final labels, similar to that described in the studies by Nakov et al [90] and Rosenthal et al [91]. First, if all 3 annotators agreed on a label (full agreement), that label was accepted. Second, if 2 of the 3 agreed on a label (partial agreement), that label was also accepted. Third, if there was no agreement, the label was set as neutral (no agreement). Fleiss κ measure was calculated to assess the agreement between the 3 annotators of each test data set. The associated *P* values were computed to test if the agreement between annotators was substantially better than what would be expected by chance. Further details of the training and test data sets are provided in Table 2. Pearson correlation coefficients were also calculated to evaluate the degree of agreement between each pair of annotators, as shown in Figure 2.

**Table 2:** Details of the National Institutes of Health (NIH) and Stanford data sets.

	Training (n=130)			Test (n=500)	
	NIH	Stanford	NIH		Stanford
Sentences, n (%)	130 (100)	130 (100)	500 (100)		500 (100)
Negative sentences, n (%)	71 (54.6)	45 (34.6)	223 (44.6)		234 (46.8)
Neutral sentences, n (%)	51 (39.2)	41 (31.6)	232 (46.4)		117 (23.4)
Positive sentences, n (%)	8 (6.2)	44 (33.8)	45 (9)		149 (29.8)
Full agreement, n (%)	N/A^a^	N/A	340 (68)		385 (77)
Partial agreement, n (%)	N/A	N/A	159 (31.8)		112 (22.4)
No agreement, n (%)	N/A	N/A	1 (0.2)		3 (0.6)
Fleiss κ	N/A	N/A	0.6311		0.7572
*P* value	N/A	N/A	*<*.001		*<*.001

^a^N/A: not applicable.

#### Preprocessing

The survey responses contained personal identifiable information and multiple sentences covering different themes, for example, 2020 presidential elections and COVID-19 vaccines. Therefore, preprocessing steps included splitting responses into sentences, replacing people’s names, suppressing email addresses, and lemmatizing and converting text to lower case.

### Sentiment Analysis Applications

We considered popular sentiment analysis applications available on the internet that use rules, machine learning, and fine-tuned LLMs.

#### Linguistic Inquiry and Word Count 2015

Linguistic Inquiry and Word Count 2015 (LIWC2015) [24,92,93] is a text analysis software that identifies and calculates the frequency of different categories of words in texts, such as pronouns, emotional words, cognitive words, and social words. LIWC2015 seeks to group words into categories that can be used to analyze psycholinguistic features in texts. Researchers in various fields, including psychology, sociology, and computer science, have used LIWC2015 to study a wide range of topics, such as personality, emotional expression, deception, and social interaction. LIWC2015 has also been used in various relevant studies on sentiment analysis. It provides with a summary variable “Tone” that combines positive and negative dimensions (*posemo* and *negemo*) into a single one. The higher the tone, the more positive it is. The tone ranges from 0 to 100. Numbers <50 indicate a more negative emotional tone. The default LIWC2015 Dictionary contains approximately 6400 words, word stems, and select emoticons.

#### SentiStrength

SentiStrength is a sentiment analysis tool that assigns scores to words and phrases based on their positive or negative sentiment [94-96]. It calculates an overall sentiment score for the text by combining these individual scores. This tool can provide dual-, binary-, trinary-, or single-scale results. In this study, a single scale ranging from −4 (extremely negative) to 4 (extremely positive) was chosen, with 0 indicating neutral sentiment. SentiStrength uses linguistic and lexicon-based methods. Linguistic methods involve rules and heuristics for identifying sentiment-bearing words and phrases, including cues such as repeated punctuation, emoticons, negations, and capital letters. The lexicon used consists of 2546 terms associated with polarity and intensity. Part of the lexicon was added from General Inquirer, including word roots such as “extrem*” to recognize variants. Training data sets included posts from various platforms such as BBC Forum, Twitter, YouTube, Digg.com, MySpace, and Runners World.

#### TextBlob

TextBlob is a Python library used in NLP tasks [11,74], such as part-of-speech tagging, sentiment analysis, and noun phrase extraction. TextBlob outputs a polarity score ranging from −1 to 1. A negative score signifies a negative sentiment, a positive score indicates a positive sentiment, and a score of 0 represents a neutral sentiment. TextBlob includes 2 analysis approaches: a rule-based model and a supervised machine learning naïve Bayes classifier model.

#### VADER

VADER [12,97] is a rule-based model designed for analyzing sentiment in social media text. It uses 5 rules based on grammatical and syntactical patterns to determine sentiment intensity. These rules involve punctuation, capitalization, degree modifiers, conjunctions such as “but,” and trigram evaluation to identify negations that can affect polarity. VADER was developed and validated using a gold standard list of lexical features, including LIWC, General Inquirer, and Affective Norms for English Words. The model was trained on various data sets, including tweets, New York Times opinions, movie reviews, and Amazon product reviews.

#### Stanza

Stanza is an open-source Python library that provides several methods for performing NLP tasks [13,98], including part-of-speech tagging, named entity recognition, dependency parsing, and sentiment analysis. Stanza’s sentiment analysis module assigns a positive, negative, or neutral sentiment score (0, 1, or 2, respectively) to each sentence in a given text. Stanza’s sentiment analysis tool is based on a convolutional neural network model using the vectors trained by Mikolov et al [99] on 100 billion words from Google News as well as a combination of lexical and syntactic features. It was trained on large data sets including movie reviews and the Stanford Sentiment Treebank. Unlike other methods, Stanza includes preprocessing of its own (sentence splitter and tokenizer).

#### TweetEval

TweetEval is a benchmarking platform for Twitter-specific classification tasks [100]. TweetEval consists of 7 NLP tasks: irony detection, offensive language detection, emoji prediction, emotion recognition, hate speech detection, stance detection, and sentiment analysis. Using TweetEval, a common set of evaluation metrics and data set, researchers and practitioners can compare the performance of different models on the same tasks and identify the most effective models for different NLP applications. TweetEval provides a leaderboard for ranking the performance of different models on the sentiment analysis task. The leaderboard is based on the F_1_-score. TweetEval returns 3 labels (positive, negative, and neutral) associated with a weight. TweetEval sentiment analysis is based on the RoBERTa model, an LLM based on BERT (trained on 58M tweets), and fine-tuned on the SemEval 2017 sentiment analysis data set (approximately 40,000 tweets) [91].

#### Pysentimiento

Pysentimiento is an open-source Python library that includes models for sentiment analysis and social NLP tasks, such as hate speech detection, irony detection, emotion analysis, named entity recognition, and part-of-speech tagging, in several languages such as English, Spanish, Portuguese, and Italian [101,102]. The English model for sentiment analysis is based on BERTweet [103], a RoBERTa model, trained on English tweets and also fine-tuned on the SemEval 2017 sentiment analysis data set [91]. Pysentimiento returns 3 polarity labels per text associated with a weight.

#### NLPTown

NLPTown [104] is a sentiment analysis application based on a BERT-base-multilingual-uncased model, fine-tuned for sentiment analysis on product reviews for 6 languages (English, Dutch, German, French, Spanish, and Italian), and predicts the sentiment of the review as the number of stars (1-5).

### Few-Shot Learning With Open Pre-Trained Transformers Language Models

As mentioned previously, few-shot learning seeks to address the challenge of sentiment analysis when only a small amount of labeled data is available for training. In traditional supervised learning, models are trained on large data sets with many labeled examples. However, in some applications such as sentiment analysis, labeled survey data are scarce or expensive to obtain, making it difficult to train accurate models. In this study, we used the Open Pre-Trained Transformers (OPT) [105], a suite of decoder-only pre-trained transformers ranging from 125M to 175B parameters created by Meta AI. OPT has been used in several applications but has never been applied to sentiment analysis. This model has shown to perform similarly to the GPT-3 [60] on several NLP tasks. The OPT model was built using a data set of 180B tokens. This represents approximately 23% (180B/780B) of the amount of data set tokens used for the Pathways Language Model [52]. The largest OPT model has comparable number of parameters to GPT-3 (175B parameters) [60], although we used all models except for the latter given graphics processing unit limitations. The novelty of OPT is its availability as open source (albeit only for academic research).

### Zero-Shot Learning With ChatGPT

Zero-shot learning refers to the use of a model to perform a task for which it has not been explicitly trained. Thus, zero-shot learning for sentiment analysis recognizes and classifies sentiment in text without being explicitly provided with examples of sentiment labels. Instead, the model is trained on related tasks, such as language modeling or machine translation, which enables it to understand the underlying structure of the language and the context in which it is used. In this study, we used ChatGPT (based on GPT-3.5), which has significantly improved the performance of several NLP tasks. GPT-3.5 is a model with 175B parameters created by OpenAI and trained on a vast amount of text data sourced from the internet using both reinforcement and supervised learning techniques. For this paper, we generated a polarity score for each sentence *x* by asking ChatGPT “What is the sentiment of the following sentence ‘x.’”

### Ethical Considerations

The NIH survey was approved by the Institutional Review Board of the NIH (reference number 20MN085), and all participants provided consent for the study. The Stanford survey was approved by Stanford’s Institutional Review Board (reference number 55436), and all participants provided consent for the study.

All survey data and responses in both the NIH and the Stanford data sets were anonymized and associated with a unique ID. Participants from both studies were not compensated for participating in the surveys.

## Results

### Evaluation Metrics

To assess the overall performance of the sentiment analysis tools, we evaluated the accuracy, macro *F*-measure, macro precision, and macro recall. Macro evaluation metrics were recommended in the NLP competition SemEval-2017 Task 4 [91].

### Preparation of Applications for Evaluation

#### Harmonization of Applications’ Outputs

The LIWC2015, Stanza, and SentiStrength applications produce outputs that are measured on distinct scales. LIWC2015 generates a continuous value ranging from 0 to 100; SentiStrength generates an integer score ranging from *−*4 to 4; and Stanza produces a discrete whole number score of 0, 1, or 2, which correspond to negative, neutral, and positive sentiments, respectively. Therefore, it is necessary to convert these scores to a common range of [*−*1*,* 1], as formally defined in equation 1.


score’(x) = 2 × (score[x] − score[x]_min_) / (score[x]_max_ − score[x]_min_) − 1 **(1)**

**Figure 3:**
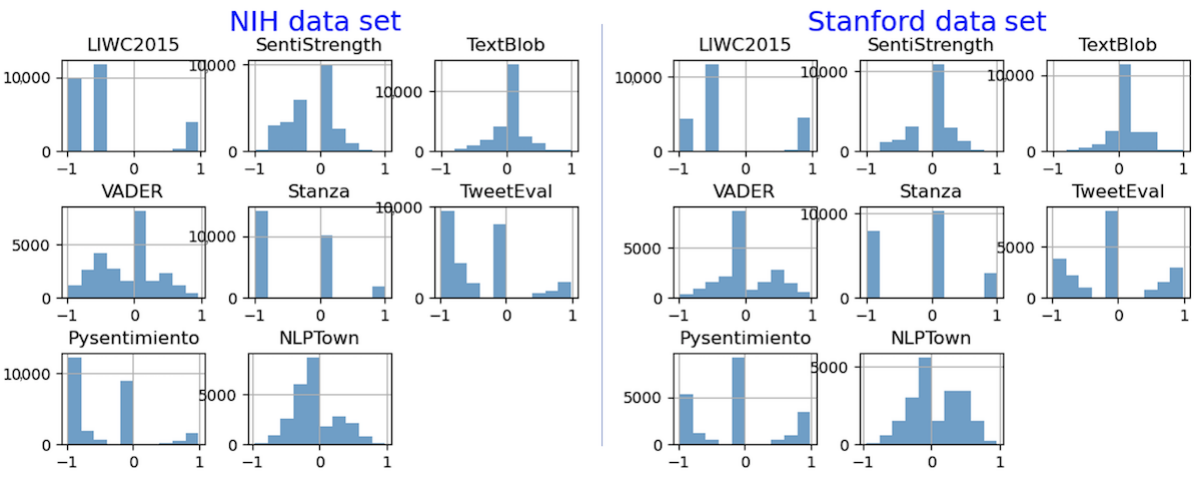
Distribution of sentiment scores across all applications on the National Institutes of Health (NIH) and Stanford data sets. LIWC2015: Linguistic Inquiry and Word Count 2015; VADER: Valence Aware Dictionary and Sentiment Reasoner.

The distribution of sentiment scores across all tools is shown in Figure 3. We then classify all negative values as negative sentiment, all 0 values as neutral, and all positive values as positive sentiment. It is important to note that the VADER application uses a slightly different classification approach, considering a score ≤0.05 to be negative, a score between −0.05 and 0.05 to be neutral, and a score ≥0.05 to be positive.

#### Fine-Tuning for Few-Shot Learning

We used few-shot learning using our small amount of training data to fine-tune the OPT models, rather than training them from scratch. For this experiment, the training data set was split into 85% (110/130) for feeding the model and 15% (20/130) for validation. Given the memory constraints, we considered only OPT 125M, 350M, 1.3B, and 2.7B. We performed a hyperparameter search to optimize the performance of the model on sentiment analysis. We considered learning rate=[3×10*^−^*^4^, 1×10*^−^*^4^, 3×10*^−^*^5^, and 1×10*^−^*^5^], batch size=[4, 8, 16, and 32], number of epochs from 1 to 7, and the AdamW optimizer. The models that performed the best were OPT-1.3B and OPT-2.7B, using a learning rate of 1×10*^−^*^5^, a batch size of 32, and 5 epochs. These were the models used to obtain the test set results reported in next subsections.

### Experiment 1: Correlation Between the Outputs of Applications

**Figure 4:**
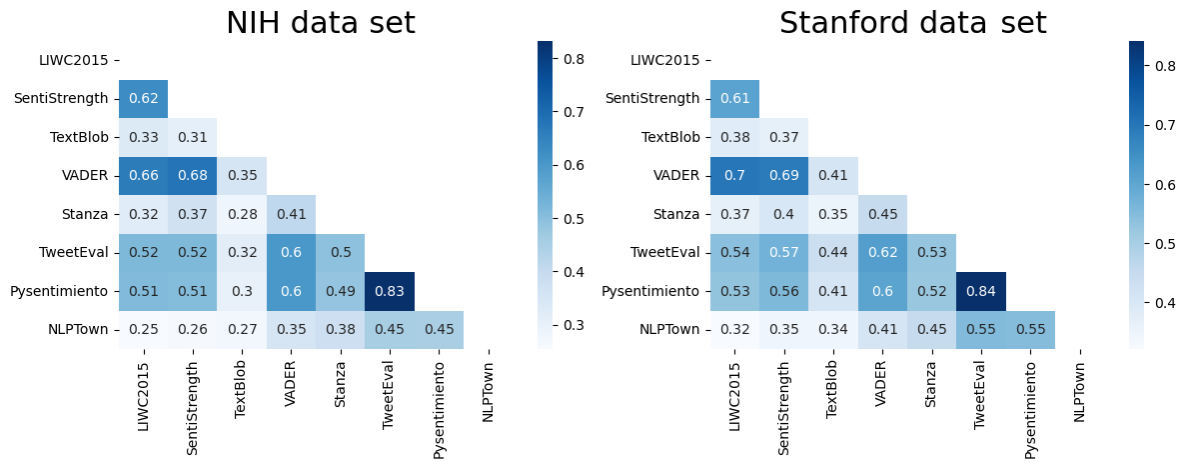
Pearson correlation matrix of score applications on the National Institutes of Health (NIH) and Stanford data sets. LIWC2015: Linguistic Inquiry and Word Count 2015; VADER: Valence Aware Dictionary and Sentiment Reasoner.

The objective was to evaluate the agreement level among various methods for predicting the sentiments of COVID-19 survey responses. Understanding the methods’ agreement or divergence was crucial in determining the reliability and accuracy of predictions, allowing for accurate studies of the relationship between language use and mental health. The Pearson correlation coefficient was used to assess the reliability of the tools, as shown in Figure 4. Disagreement among the methods prompted us to evaluate few-shot learning to obtain high-quality predictions.

### Experiment 2: Prediction of Sentiment Scores

Tables 3 and 4 show the performance results obtained by all applications, few-shot learning, and zero-shot learning techniques on the NIH and Stanford test data sets, respectively. Both test sets comprised 500 sentences each, as detailed in the *Data* section. The top 2 performance results are italicized. Of note, a perfect classifier that accurately categorizes all items obtains a value of 1, whereas a perverse classifier that misclassifies all items achieves a value of 0. However, a trivial classifier that assigns all sentences to the same category (positive, negative, or neutral) and a random classifier both have a value of 0.3333.

ChatGPT achieved a significant improvement in sentiment analysis compared with other models through zero-shot learning. On the NIH data set, ChatGPT outperformed few-shot learning (OPT-1.3B and OPT-2.7B) by 6% in accuracy and 7% in *F*-measure. Similarly, on the Stanford data set, ChatGPT showed better results than the OPT-1.3B and OPT-2.7B models, with 6% higher accuracy and 4% higher *F*-measure.

Moreover, to further evaluate the sentiment analysis tools, we used Bayesian analysis, as recommended by Benavoli et al [106], to assess the statistical significance of the performance of the methods. Specifically, we applied the Bayesian signed-rank test [107] to compare the accuracies achieved across multiple data sets. This test quantifies the likelihood of observing the signed ranks of accuracy differences under both the null hypothesis (indicating no significant difference) and alternative hypothesis (indicating a significant difference). The Bayesian signed-rank test is designed to compare performance over multiple data sets (≥2); therefore, we further partitioned the independent Stanford and NIH data sets. Each data set was partitioned into 3 subsets, based on the sentiment label assigned to them, resulting in positive, neutral, and negative subsets for each data set.

**Figure 5:**
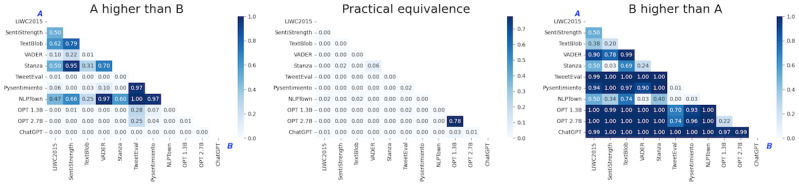
Bayesian analysis conducted on accuracy performances of 11 sentiment analysis methods across 6 different subsets. LIWC2015: Linguistic Inquiry and Word Count 2015; OPT: Open Pre-Trained Transformers; VADER: Valence Aware Dictionary and Sentiment Reasoner.

This division was influenced by insights from our prior analysis, which highlighted inherent distinctions among sentences associated with positive, neutral, and negative labels. For instance, positive sentences exhibited a preponderance of positive adjectives, whereas negative sentences featured more negative adjectives, and neutral sentences tended to emphasize facts that are characteristic of the neutral category. Therefore, we assumed a degree of independence across subsets within each data set. The heat map diagram in Figure 5 shows the results of our Bayesian analysis, with cells corresponding to row *i* and column *j*. On the left side, “A higher than B” indicates the probability that method *i* performs better than classifier *j*. The center indicates the probability of practical equivalence between methods *i* and *j*. Similarly, on the right side, “B higher than A” indicates the probability that method *j* is better than classifier *i*. These experiments confirmed that ChatGPT performed better than all the other alternatives. The OPT models showed similar performance to methods other than ChatGPT and could be considered as a viable second option.

**Table 3:** Results on the National Institutes of Health (NIH) test data set.

Application	Precision	Recall	*F*-measure	Accuracy
LIWC2015^a^	0.2733	0.5226	0.3587	0.4540
SentiStrength	0.5732	0.6006	0.5814	0.6480
TextBlob	0.4505	0.4776	0.4053	0.4340
VADER^b^	0.6302	0.7036	0.6097	0.6580
Stanza	0.6178	0.5758	0.5886	0.6300
TweetEval	0.7818	*0.8318* ^*c*^	0.7898	0.7840
Pysentimiento	0.7738	0.7780	0.7699	0.7760
NLPTown	0.4338	0.5173	0.4210	0.4520
OPT^d^ 1.3B (few-shot)	0.8032	0.8000	0.7992	0.8000
OPT 2.7B (few-shot)	*0.8061*	0.8040	*0.8050*	*0.8040*
ChatGPT (zero-shot)	*0.8526*	*0.8926*	*0.8668*	*0.8600*
All negative	0.1487	0.3333	0.2056	0.4460
All neutral	0.1547	0.3333	0.2113	0.4640
All positive	0.0300	0.3333	0.0550	0.0900

^a^LIWC2015: Linguistic Inquiry and Word Count 2015.

^b^VADER: Valence Aware Dictionary and Sentiment Reasoner.

^c^Italicization represents the top 2 performance results.

^d^OPT: Open Pre-Trained Transformers.

**Table 4:** Results on Stanford test data set.

Application	Precision	Recall	*F*-measure	Accuracy
LIWC2015^a^	0.3752	0.4391	0.3890	0.5400
SentiStrength	0.5738	0.5561	0.5335	0.5420
TextBlob	0.4757	0.4872	0.4527	0.4600
VADER^b^	0.5875	0.5919	0.5755	0.5840
Stanza	0.5975	0.4987	0.4859	0.5040
TweetEval	0.7366	0.7178	0.7090	0.7200
Pysentimiento	0.6731	0.6362	0.6267	0.6440
NLPTown	0.5163	0.5192	0.5056	0.5420
OPT^c^ 1.3B (few-shot)	*0.8323* ^*d*^	*0.8160*	*0.8211*	*0.8160*
OPT 2.7B (few-shot)	0.8288	0.8100	0.8147	0.8100
ChatGPT (zero-shot)	*0.8632*	*0.8779*	*0.8662*	*0.8740*
All negative	0.1560	0.3333	0.2125	0.4680
All neutral	0.0780	0.3333	0.1264	0.2340
All positive	0.0993	0.3333	0.1531	0.2980

^a^LIWC2015: Linguistic Inquiry and Word Count 2015.

^b^VADER: Valence Aware Dictionary and Sentiment Reasoner.

^c^OPT: Open Pre-Trained Transformers.

^d^Italicization represents the top 2 performance results.

## Discussion

### Principal Findings

Our primary objective was to assess various sentiment analysis tools for the purposed of predicting the sentiments of survey responses during the COVID-19 pandemic. Obtaining a thorough understanding of the tools’ degree of agreement, as shown in Figure 4, was crucial for determining whether they could be used as surrogates for human labeling. The disagreement between tools led us to try ensemble methods to produce more reliable ratings. Fine-tuned BERT models such as TweetEval and Pysentimiento outperformed the other baseline methods. Fine-tuned methods have the ability to learn domain-specific patterns from text, resulting in better performance than lexicon- and rule-based methods. However, these techniques often require large training data sets to achieve optimal performance, such as the 40k tweet data set used to train TweetEval and Pysentimiento.

As part of the process of determining agreement between tools, we labeled a small data set (260 sentences), which is what prompted us to consider the possibility of using few-shot and zero-shot learning techniques. We then investigated the performance of OPT, which is unexplored in sentiment analysis, for few-shot learning using a small training data set (260 sentences). The OPT-1.3B and OPT-2.7B models surpassed all the baseline methods as well as the fine-tuned BERT models. This highlighted the potential of few-shot learning in dealing with scarce annotated data and the effectiveness of few-shot learning. Although better results could have been achieved with a larger training set, these experiments primarily aimed to investigate the potential of OPT using limited annotated data. The potential is to be able to produce models tailored to specific research applications, with only a small time investment by domain experts. We believe that these models can significantly contribute to the sentiment analysis of health- and clinical-related surveys and can be further fine-tuned with additional data and optimized hyperparameters.

Our investigation also encompassed zero-shot learning with ChatGPT, which exhibited remarkable performance compared with all other models, including few-shot learning with OPT, as presented in Tables 3 and 4. Note that GPT-3.5—the model behind ChatGPT—is trained on related tasks, such as language modeling or machine translation. This enabled it to understand the underlying structure of sentiment-related language and the context in which it is used. Moreover, the necessity for manual text annotations in sentiment analysis tasks makes ChatGPT and other LLMs particularly attractive. As demonstrated by Ziems et al [108], LLMs can alleviate the workload of human annotators in a zero-shot manner, thereby enhancing the efficiency of social-science analysis. In addition, a study [109] found that ChatGPT outperformed crowd workers in various text annotation tasks, including assessing relevance, stance, topics, and frame detection. These findings suggest that there may be potential in using ChatGPT and other recent LLMs for annotation in clinical NLP and reserving human input for quality control. Sentiment analysis tools based on LLMs, such as ChatGPT, automatically identify relevant features, reducing the need for manual engineering, which is a common requirement in tools such as LIWC 2015 and VADER. In addition, LLMs enable fine-tuning, allowing for potential adaptation to different sentiment analysis tasks (eg, in new domains) without the need for complete retraining. LLM-based tools can also capture longer-range context for more accurate sentiment assessment.

### Limitations

There exist several limitations and risks of ChatGPT and other non–open-source LLMs regarding protected health information (PHI). Non–open-source LLMs require sending information to an external server and do not provide transparency into how they handle PHI, making it difficult to assess how the model is processing and protecting sensitive information. They may also have security vulnerabilities that can be exploited to gain unauthorized access to PHI. Note also that LLMs are not specifically designed for sentiment analysis, which may sometimes lead to errors, for instance, subtle sarcasm such as “Oh yes, great job!,” context-dependent negation as in “The vaccine was not as bad as I thought,” and idiomatic expressions such as “It’s a piece of cake.” They may encounter difficulties with nuanced health-related terminology and concepts. Therefore, specialized health terminology may require additional adaptation beyond general text fine-tuning, for instance, medical abbreviations and acronyms such as “The patient teared up because of a significant increase in their CD4 count” and “So, my mom’s HbA1c levels have improved after insulin therapy.” In addition, although several outputs may sound plausible, they may occasionally be incorrect. In our view, the output of LLMs should not be used without a plan for human quality control (eg, via sampling) or mitigation (eg, repeated validation). This is crucial for ensuring the accuracy and reliability of the generated content, as LLMs may produce results that require refinement or correction before dissemination. Moreover, there are constraints on the ability to access ChatGPT via its application programming interface, and this may make it too costly or time-consuming to do so. Therefore, researchers and health care practitioners might also opt to use an open-source language model for their NLP-related projects, such as OPT, which can be run on site and perform well on sentiment analysis.

Finally, our study focused on using surveys to understand people’s feelings, specifically regarding COVID-19, which was a very important topic at the time. Thus, our conclusions apply specifically to discussions about COVID-19 and may not be true for other subjects. In addition, it is important to highlight that the Stanford data set has an implicit polarity bias: it specifically asks for positive effects (“Although this is a challenging time, can you tell us about any positive effects or ‘silver linings’ you have experienced during this crisis?”) and difficulties (“Have you experienced any difficulties due to the coronavirus crisis?”). The NIH data set poses a single, less-biased question. Therefore, it is crucial to be careful when generalizing our findings beyond the scope of COVID-19 during the studied time frame.

## Abbreviations

BERT: Bidirectional Encoder Representations from Transformers
DNN: deep neural network
ELMo: Embeddings from Language Models
GPT: Generative Pre-trained Transformers
LIWC: Linguistic Inquiry and Word Count
LIWC2015: Linguistic Inquiry and Word Count 2015
LLM: large language model
NIH: National Institutes of Health
NLP: natural language processing
OPT: Open Pre-Trained Transformers
PaLM: Pathways Language Model
PHI: protected health information
RoBERTa: Robustly optimized Bidirectional Encoder Representations from Transformers approach
VADER: Valence Aware Dictionary and Sentiment Reasoner

## Data Availability

The test data sets generated and analyzed during this study are deidentified and freely available in the FigShare repository [89]. The source code for fine-tuning the OPT models and using ChatGPT in the experiments conducted in this study is publicly accessible on GitHub [110].
